# Reflections of patient and public involvement from a commissioned research project evaluating a nationally implemented NHS programme focused on diabetes prevention

**DOI:** 10.1186/s40900-023-00447-0

**Published:** 2023-06-14

**Authors:** Rhiannon E. Hawkes, Caroline Sanders, Claudia Soiland-Reyes, Lisa Brunton, Kelly Howells, Sarah Cotterill, Carole Bennett, Eric Lowndes, Manoj Mistry, Helen Wallworth, Peter Bower

**Affiliations:** 1 Division of Psychology and Mental Health, Faculty of Biology, Medicine and Health, School of Health Sciences, Manchester Centre of Health Psychology, University of Manchester, Manchester, UK; 2 Division of Population Health, Health Services Research & Primary Care, Faculty of Biology, Medicine and Health, NIHR Greater Manchester Patient Safety Translational Research Centre (NIHR PSTRC), Centre for Primary Care and Health Services Research, School of Health Sciences, University of Manchester, Manchester, UK; 3 Research & Innovation, Northern Care Alliance NHS Foundation Trust, Salford, UK; 4 North West Ambulance Service NHS Trust, Bolton, UK; 5 Division of Population Health, Health Services Research & Primary Care, Faculty of Biology, Medicine and Health, NIHR Applied Research Collaboration Greater Manchester (NIHR ARC GM), Centre for Primary Care and Health Services Research, School of Health Sciences, University of Manchester, Manchester, UK; 6 Division of Population Health, Health Services Research & Primary Care, Faculty of Biology, Medicine and Health, Centre for Biostatistics, School of Health Sciences, University of Manchester, Manchester, UK; 7 DIPLOMA Patient and Public Involvement Group, University of Manchester, Manchester, UK

**Keywords:** Patient and public involvement, Reflections, Diabetes prevention, Type 2 diabetes, National programmes, Evaluation programmes

## Abstract

Patient and Public Involvement and Engagement (PPIE) in research is recognised by the National Institute for Health and Care Research as crucial for high quality research with practical benefit for patients and carers. Patient and public contributors can provide both personal knowledge and lived experiences which complement the perspectives of the academic research team. Nevertheless, effective PPIE must be tailored to the nature of the research, such as the size and scope of the research, whether it is researcher-led or independently commissioned, and whether the research aims to design an intervention or evaluate it. For example, commissioned research evaluations have potential limits on how PPIE can feed into the design of the research and the intervention. Such constraints may require re-orientation of PPIE input to other functions, such as supporting wider engagement and dissemination. In this commentary, we use the ‘Guidance for Reporting Involvement of Patients and the Public’ (GRIPP2) short form to share our own experiences of facilitating PPIE for a large, commissioned research project evaluating the National Health Service Diabetes Prevention Programme; a behavioural intervention for adults in England who are at high risk of developing type 2 diabetes. The programme was already widely implemented in routine practice when the research project and PPIE group were established. This commentary provides us with a unique opportunity to reflect on experiences of being part of a PPIE group in the context of a longer-term evaluation of a national programme, where the scope for involvement in the intervention design was more constrained, compared to PPIE within researcher-led intervention programmes. We reflect on PPIE in the design, analysis and dissemination of the research, including lessons learned for future PPIE work in large-scale commissioned evaluations of national programmes. Important considerations for this type of PPIE work include: ensuring the role of public contributors is clarified from the outset, the complexities of facilitating PPIE over longer project timeframes, and providing adequate support to public contributors and facilitators (including training, resources and flexible timelines) to ensure an inclusive and considerate approach. These findings can inform future PPIE plans for stakeholders involved in commissioned research.

**Supplementary Information:**

The online version contains supplementary material available at 10.1186/s40900-023-00447-0.

## Abstract (highlights)

This commentary uses the ‘Guidance for Reporting Involvement of Patients and the Public’ checklist to describe the activities and lessons learned from the work of a Patient and Public Involvement and Engagement (PPIE) group working with researchers evaluating the National Health Service (NHS) Diabetes Prevention Programme (a nine-month behavioural support programme offered to adults who are at risk of developing type 2 diabetes). The evaluation aimed to find out if the NHS Diabetes Prevention Programme was meeting its aim of reducing type 2 diabetes whilst offering value for money. The PPIE group was made up of members of the public who were either living with diabetes, at risk of diabetes, or had a family history of diabetes. Their aim was to support the work of the research team to identify research priorities, design the research, clarify the meaning of the research results, and help communicate the results to the wider public. However, given that the programme was an NHS service already being delivered in practice, there were limits to what the PPIE group could do in terms of designing the intervention. Instead, our public contributors were involved in all stages of conducting the research, with greater focus on communication of findings from the research evaluation. This report provides an opportunity to reflect on PPIE in a longer-term evaluation of a national programme and reflect on involvement in dissemination and policy impact. We hope that future evaluation programmes can learn from our experiences and optimise PPIE for similar national evaluations.

**Supplementary Information:**

The online version contains supplementary material available at 10.1186/s40900-023-00447-0.

## Background

### Patient and public involvement and engagement

Patient and Public Involvement and Engagement (PPIE) is defined by INVOLVE (now known as the National Institute for Health and Care Research [NIHR] Centre for Engagement and Dissemination) as “research carried out ‘with’ or ‘by’ members of the public who are actively involved in the research projects” [1]. Public involvement is now an essential requirement for research funded by NIHR in the United Kingdom (UK) and for funders globally [2–4], to ensure that research is of high quality and of practical benefit [1].

Involving members of the public in research improves both quality and relevance of the research. They provide both personal knowledge and experiences of using a service or living with a health condition, which complements that of the research team [1, 5]. In addition to being adequately supported financially, effective PPIE needs to be tailored to the nature of the research, such as the size and scope of the research, whether the research is researcher-led or independently commissioned by a research funder, and whether the research aims to design an intervention or evaluate it. For example, independent commissioned research evaluations may have limits on how PPIE can inform the design of the intervention when it is already implemented in routine practice. Thus, ensuring a PPIE strategy is in place is important for all types of health research, including diabetes [6].

We reflect on PPIE work conducted as part of an independent commissioned research evaluation called ‘DIPLOMA’ (Diabetes Prevention Long-term Multimethod Assessment) evaluating the NHS Diabetes Prevention Programme (see Box 1 for more information about the NHS Diabetes Prevention Programme and Box 2 for more information about DIPLOMA). The DIPLOMA PPIE group is the focus of this commentary, which has been co-authored with members of the group. The PPIE members’ experiences are included throughout the article and are reflected using the terms ‘we’ and ‘our’.

**Box 1:** The NHS Diabetes Prevention Programme

Type 2 diabetes is a global public health priority [7], and 13.6 million people in the UK are now at an increased risk of developing the condition [8]. Type 2 diabetes is preventable by behavioural modifications such as improved diet, increased physical activity and weight loss. Prevention programmes implemented worldwide provide behaviour change interventions for individuals at risk. Trials and real-world studies have found such programmes to be effective at promoting weight loss and thereby reducing the risk of type 2 diabetes [9].
In response to this, the NHS Diabetes Prevention Programme was launched in 2016; a nine-month behaviour change intervention for adults in England who have been identified as having elevated blood glucose levels and therefore at an increased risk of developing type 2 diabetes [10]. The programme is commissioned by NHS England and provides education and support on how to improve diet and increase physical activity to achieve weight loss. The NHS Diabetes Prevention Programme is the largest in the world to achieve universal national coverage [11].

**Box 2:** The DIPLOMA research project

The ‘DIPLOMA’ research project (Diabetes Prevention Long-term Multimethod Assessment) is a commissioned mixed-methods study delivered by a multidisciplinary team, designed to provide a rigorous longer-term assessment of the NHS Diabetes Prevention Programme to ensure it is meeting the aim of reducing type 2 diabetes in England in a way that is sustainable and cost-effective [12]. This programme of research was funded by NIHR in 2017 and consisted of eight work packages to evaluate: access and equity of those who enter the NHS Diabetes Prevention Programme, implementation of the programme across England, service delivery and fidelity of the programme, patient outcomes within the programme, and long-term cost effectiveness. As part of the PPIE strategy, DIPLOMA researchers were keen to involve public contributors from the outset. We wanted to involve those who were living with diabetes, were at risk of diabetes, or had a family history of diabetes, and could provide valuable insights on how to design, implement and disseminate research findings to members of the public in a way that was accessible and engaging.	

### Focus of this commentary

This commentary uses the ‘Guidance for Reporting Involvement of Patients and the Public’ (GRIPP2) short form checklist, a tool for reporting of PPIE in health and social care [13], and applies it to PPIE in the DIPLOMA research project. GRIPP2 was developed to enhance the quality and transparency of the evidence base for PPIE, so that readers can learn from PPIE in other studies and understand what the result of this involvement was [13]. The short checklist encourages research teams to report five topics: aims, methods, study results, discussion, and reflections of PPIE in research (Additional File 1). We reflect on what went well and the challenges we faced, so that others can learn from our experiences. In particular, we reflect on the challenges and lessons learned in facilitating PPIE involvement in commissioned research for a longer-term evaluation of a programme that was already implemented as an NHS service.

We also reflect on collaboration in terms of recently published UK standards for effective PPIE [14]. These six standards list a clear set of statements for effective public involvement and encourages behaviours such as flexibility, sharing and learning, and respect for others. Two of the UK standards that are particularly relevant in relation to research evaluations closely connected to national policy are ‘communications’ and ‘impact’ [14]. We hope that other researchers, members of the public and research funders can learn from our experiences and optimise public involvement for similar national evaluations.

## Public involvement and engagement in the DIPLOMA research project reported using GRIPP2

### Aim

The aim of the PPIE involvement group in the DIPLOMA research project was for the research team to work collaboratively with public contributors to identify research priorities, design the research, analyse and interpret the results and disseminate research findings to lay audiences, ensuring that patients and the public were informed about ongoing developments throughout the life of the DIPLOMA evaluation. In addition to the PPIE group, DIPLOMA recruited two lay members to our Study Steering Committee (SSC) group, whose role was, together with external clinicians and expert researchers, to provide overall supervision for the project on behalf of the NIHR [15]. The work of the SSC lay members is not part of this commentary.

### Methods

#### Pre-funding: identifying research priorities

Prior to funding for DIPLOMA, patients and the public were actively involved in preparing the application for this programme of research. An advert was circulated via an existing PPIE group and via a PPIE network. The research team recruited six people (living with diabetes, at risk, or with family history), varying in age, gender, ethnicity and disabilities. Researchers provided a summary of the research plans and asked what they would like to know about the effectiveness of the NHS Diabetes Prevention Programme, services, organisations and participants. Many responses mapped onto the team’s existing questions, including: effect on diabetes prevention and other health outcomes; cost effectiveness; equal access; and understanding implementation and service delivery. New topics were suggested that were added to potential research plans: choice of service, and ability to cope with the risk of diabetes; the impact of wider social networks on uptake; change in GP referrals over time; take-up by people with new diagnosis or established conditions; and clarity of information provided at time of referral.

#### Post-funding: involvement in the DIPLOMA research project

PPIE in the DIPLOMA project is summarised in Table 1. Five of the six public contributors involved in developing the research priorities pre-funding continued their involvement when DIPLOMA was funded. The involvement of public contributors in the NHS Diabetes Prevention Programme design was somewhat limited given that (a) this was a commissioned research evaluation where funders set out a broad outline to answer specific research questions, and (b) the NHS Diabetes Prevention Programme was already implemented in routine practice. Since the aim of DIPLOMA was to provide recommendations for the wider implementation of the programme to improve outcomes for patients, we therefore planned to have an equal focus on public ‘engagement’ to disseminate the research findings to members of the public throughout the DIPLOMA project, keeping members of the public informed of the findings through blogs, videos and lay summaries. DIPLOMA had therefore budgeted for engagement activities throughout the duration of the research, an example of a best practice activity to support stakeholder engagement [16].

**Table 1:** Involvement of Public Contributors in the DIPLOMA Research Project

**Public contributors**
A DIPLOMA PPIE group was formed in 2017; there have been a total of 10 contributors throughout the DIPLOMA evaluation, and five who have regularly contributed since 2020 PPIE members were recruited either from: (a) a previous PPIE group who expressed an interest in the DIPLOMA project due to personal and family experiences, (b) a PPIE network called ‘Research for the Future’, a collaboration between NIHR Clinical Research Network: Greater Manchester, North West EHealth, Northern Care Alliance NHS Group and Health Innovation Manchester, or (c) Diabetes UK Contributors included representation from people at risk of type 2 diabetes, people with a family history of diabetes, and people from different backgrounds including ethnic minorities Two lay members were also recruited to our Study Steering Committee group, alongside clinicians and researchers outside of the research team
**Meetings**
Members of the research team facilitated the PPIE group throughout the five-year project. Everyone was considered equal The PPIE group met 18 times in total (initially face-to-face, and then virtually during and following the Covid-19 pandemic)
**Payments**
All PPIE members were paid for their involvement, in line with current guidance [17] PPIE members were reimbursed for any travel expenses and for their time, including meeting attendance and any feedback activity undertaken throughout the research evaluation, as per the current guidance [17]
**PPIE involvement**
During these meetings, and via email and telephone communication in between meetings, members helped to design research studies within the different work packages, interpret research findings, and co-produce public engagement materials to disseminate research findings for lay audiences

### Study results

PPIE contribution was specifically for the DIPLOMA project, rather than for the NHS Diabetes Prevention Programme, as the service had already been developed and was implemented in routine practice at the time of the research evaluation. Therefore, public contributors were involved during all phases of the research, from the design and preparation of studies, conduct and implementation, and dissemination of research findings [15].

#### Design of research studies

Prior to the start of DIPLOMA, the research team worked with six members of the public in writing the funding bid: they raised new ideas about what research questions we should ask, advised the team about how to involve people in the study, and suggested the important role the public can have in engagement. Once the PPIE group was set up, members provided input on the wording of study materials including, but not limited to: topic guides, consent forms, information sheets and study questionnaires to ensure these were understandable and written in Plain English. Members also helped the researchers identify other areas of exploration to ask during participant interviews. For example, for one of the research topic guides the group suggested that it would be beneficial to ask the interviewees if they felt their expectations of the NHS Diabetes Prevention Programme had been met, and the group also highlighted that interviewees may need breaks factored into the interview. Researchers have also explored recruitment procedures with the PPIE group; we have provided suggestions for strategies such as approaching charities and places of worship, displaying posters in GP surgeries, and posting on social media platforms and specific online groups. As public contributors, being involved early during the research studies ensured that the research was acceptable to potential participants, and is considered a best practice approach [16].

#### Data analysis and interpretation

Some of our PPIE group have been involved in early stages of qualitative data analysis, for example, reading through a sample of anonymised transcripts of healthcare professional referrals of patients onto the programme, and providing their views on developing themes. Members of our group have also commented on quantitative results from studies, which can be more difficult to convey to a lay audience. For example, researchers asked the PPIE group on their interpretations of quality-of-life data from the NHS Diabetes Prevention Programme.

Researchers from all DIPLOMA work packages have presented findings to the PPIE group at various stages of the evaluation, and we have provided feedback and further interpretation of the findings. Members have fed back on the terminology used and some of the graphs which were too technical for lay members to understand initially. The researchers produced alternative presentation formats, which the PPIE group fed back on. This regular dialogue between the research team and PPIE group [16] ensured we were able to input on study results, provide interpretations of results, and suggest ways to present the results and complicated statistics to a wider audience.

The PPIE group have also advised on analysis plans, for example, analysis of uptake to the NHS Diabetes Prevention Programme. This dataset included data fields collected by primary care when patients registered on the programme. Some of our group highlighted that religious reasons for declining the programme and the time of year that the programme was declined (e.g., to account for religious holidays) were also important considerations for programme uptake, which was not captured in the dataset that the DIPLOMA research team had access to. In this case, the PPIE group provided valuable insight about how data collection for uptake could be optimised. However, it was not in the remit of the research team to make these direct changes and we could only feedback to commissioners.

#### Dissemination of research findings

Our PPIE group were passionate about getting involved in raising awareness about diabetes prevention. For example, we raised ideas about wider community engagement and making information as accessible as possible, especially in communities and networks where there would likely be more risks related to inequalities in diabetes and challenges for addressing this. However, it was out of the remit of the research team to ‘promote’ the NHS Diabetes Prevention Programme, although the PPIE group were able to provide valuable input for communications and dissemination of DIPLOMA findings.

Over the five-year project, we have collaborated with video producers, illustrators and scriptwriters to create a range of accessible web-based videos to promote public engagement of different aspects of DIPLOMA. This has included three animation videos describing (a) the planned DIPLOMA evaluation at the start of the project [18], (b) the overall research findings at the end of the project [19], and (c) information about making changes to health behaviours [20]. We have also co-produced two ‘Talking Heads’ videos [21, 22]; the first video summarised a qualitative study on how service users understood their type 2 diabetes risk [23], and the second video summarised research on service user take-up and experiences of the NHS Digital Diabetes Prevention Programme [24]. These have been disseminated in the public domain (e.g., via social media channels and NHS England Health Care Innovation Expo). The PPIE group co-produced the video scripts, inputted into the storyboards, and provided feedback on the overall ‘look and feel’ of the videos to ensure they were appealing and understandable for the public. We also featured in some of these videos.

The group have also co-produced lay summaries of studies, working closely with DIPLOMA researchers. This again ensured that our research findings were of wider interest to a non-academic audience. We have worked with an illustrator to produce illustrated summaries to disseminate research findings in different formats for diverse audiences. Disseminating research to the general public was particularly important for DIPLOMA, given that it was taxpayer’s money subsidising both the research and the NHS Diabetes Prevention Programme, and patients and the public have a right to influence what is supported [25]. We have provided feedback on how to optimise engagement and understanding of these materials for a lay audience, particularly commenting on use of complex statistics and acronyms. PPIE members have also written blogs about their experiences of involvement in evaluations of national programmes [26, 27].

### Discussion and conclusions

PPIE has been vital throughout the life of the DIPLOMA evaluation, from study design through to dissemination of findings. As public contributors, we have provided a unique and broader perspective on the work, which increased the potential of the DIPLOMA research to meet the needs of patients and members of the public.

However, PPIE contribution for a commissioned research evaluation of an NHS service already implemented had unique challenges. For example, there have been several occasions where the PPIE group have provided valuable feedback on the NHS Diabetes Prevention Programme, but it was not within the remit of the DIPLOMA research team to make changes to the programme itself. The purpose of the PPIE group was instead to support a research evaluation of an intervention developed by a third party, rather than supporting a research project designing the intervention; an approach much less direct than what some contributors might have been used to. The literature on public involvement has previously identified that one of the most common issues in PPIE work is unclear definition of roles and expectations of public contributors [25]; this could have been better addressed in DIPLOMA and we further reflect on this below.

Given that there was less scope for our PPIE group to provide direct feedback on the NHS Diabetes Prevention Programme itself, we were keen to give at least equal focus to ‘engagement’ as well as ‘involvement’, to produce accessible materials on findings for the public. We view this as a particular strength of our approach as this gave the PPIE a higher profile than might be the case with other research projects. Reflecting on the UK standards for PPIE [14], we were involved in developing a high number of accessible co-produced communications for a high profile policy-related national programme [18–22], demonstrating a clear focus on both ‘communications’ and ‘impact’ [14]. However, engagement work also came with its own challenges to ensure we kept a clear distinction between dissemination of the research findings and ‘promotion’ of the NHS Diabetes Prevention Programme.

Finally, national evaluations of programmes such as the NHS Diabetes Prevention Programme are often of greater complexity in terms of methods and data analyses, as was the case for DIPLOMA. For example, this research involved complex health economics and effectiveness analyses, and some public contributors found it more challenging to get involved in these quantitative components. This highlights an example of why there is a greater need for support, resources and training for both public contributors and group facilitators in large commissioned research projects such as DIPLOMA, and PPIE input on these components can take considerable time for all team members. A previous commentary on PPIE contribution in diabetes research has discussed ways in which they have achieved this, such as yearly training on research methods though interactive quizzes for patients [6]. In relation to the UK PPIE standards [14], there could have been some further learning and development opportunities identified for both PPIE contributors and the research team to help facilitate this challenge in DIPLOMA. However, the researchers very much valued the input from our PPIE group on presenting the research findings in a way that was more understandable for lay audiences, and PPIE feedback was taken on board.

### Reflections/critical perspective

Our PPIE group agreed that being involved in DIPLOMA was a positive experience where we felt listened to and our feedback was incorporated throughout the entire project; we discussed our views during a meeting specifically organised to reflect on each aspect of the research evaluation. Being part of this research team gave the opportunity for members to learn about how academic research is conducted. Importantly, it also increased our awareness of the constraints involved in both academic research and the implementation of programmes such as the NHS Diabetes Prevention Programme.

#### Lessons learned in the transition to online meetings

Partway through the project, the Covid-19 pandemic changed the way we were able to work together; it was no longer possible to meet in-person, thus we decided to use a video conferencing platform to host meetings. Although the group have missed face-to-face meetings, there have been some advantages to online meetings and this new way of working. For example, travelling to the university campus for in-person meetings was at times daunting for some members, and could take up a substantial amount of their day for a two-hour meeting. Online meetings have been more time efficient and cost-effective. Thus, the group reflected the importance of still meeting up in-person, whilst also embracing this new way of working online. Similar issues of adapting to PPIE during the pandemic have been discussed in more depth elsewhere [28].

However, this new way of working together has taught the research team some lessons which we will learn from. For example, one of our public contributors did not have access to a camera for the online meetings, which made both visual communication and feeling included difficult for this individual. It was correctly highlighted by another member of our PPIE group that such access issues would not arise in a face-to-face environment, as every effort would have been made to help members ‘get into the room’. Whilst online working was new to all of the research team during the Covid-19 pandemic, it is something we have now all become accustomed to. On reflection, this would have been an issue easily dealt with by the research team to ensure all PPIE members had equality of access and inclusive opportunities; one of the UK standards of PPIE [14]. The option to also receive some training or practice of how to use video conferencing during the transition to online meetings could have been another way to ensure the PPIE group felt included and confident in participating in online meetings and is recognised as a best practice activity for stakeholder involvement [16].

#### Challenges of being part of a longer-term research project

Our group appreciated timely and effective communication from the research team, even when all communication transferred online. However, DIPLOMA has faced its unique challenges due to the project being a lengthy and complicated programme of research, and much longer in comparison to some research projects. This resulted in a lack of continuity of researchers taking on a facilitator role over such a long programme of work, with three different PPIE research facilitators during the five-year project (REH, LB and KH), and a turnover of PPIE contributors, both of which had an impact on continuity and group dynamics. Although this was unavoidable, this had at times resulted in unintended consequences. Firstly, there was a loss of contact with original members of the PPIE group who stepped away from the group due to health issues. Secondly, newer members recruited to the PPIE group later in the project had to quickly understand and deal with the complexities of the different work packages in DIPLOMA. Although the research team provided an introduction to the DIPLOMA research when new members of the PPIE group joined, there was a lot of information for new members to take on board for a large project.

Despite DIPLOMA being a complex project with eight work packages, our group felt that the work packages had been usefully broken up to ease understanding. However, some members reported to not always comprehend where their contribution fitted into the wider project. Although during early PPIE meetings there was discussion of timelines to help show where PPIE would be sought at different time points, these had been somewhat impacted by the switch to working online. As with other policy programmes, the NHS Diabetes Prevention Programme had shifting timelines beyond the control of the research team, which made PPIE work more complex.

It was also noted that at times the duration between the meetings were too long. This led to members forgetting about some of the previous work; a particular difficulty of facilitating PPIE for a longer-term evaluation. To mitigate this, a meeting schedule could have been planned in advance for upcoming months of PPIE meetings to reduce the time between meetings when trying to find suitable dates to accommodate the group. Although the above highlights a general issue in PPIE work about desires to avoid burdening the group compared with neglecting the group [29], a more formal procedure could have been put in place for public contributors to regularly feedback any potential issues to the research team so that they could be detected earlier on during the research evaluation. For example, PPIE contribution in diabetes research has previously reported an anonymous survey to work well in obtaining feedback from the group [6].

#### Challenges of being part of a national evaluation of a commissioned NHS service

Some of our group reflected on the difficulties of PPIE contribution for a research project where the NHS Diabetes Prevention Programme was already in service, rather than an intervention being developed by the research team. It was at times frustrating for PPIE members when we were not able to have a direct impact on the design and delivery of the NHS Diabetes Prevention Programme. Although views were noted and could be fed back to commissioners, the research team had a mediating role between the PPIE group and programme developers. On reflection, the research team could have better explained at the outset to the group what was (not) in the remit of DIPLOMA to ensure better expectation management for working together [14]. The clarification of roles of public contributors in research is discussed in the literature [25, 29] and described as a best practice activity which should be established early on during the research project [15].

We also reflected on our input on the public engagement resources produced by DIPLOMA, including the blogs and videos. We discussed that DIPLOMA has been successful in informing the public via the PPIE group about the research, which were seen as examples of genuine collaboration. However, it was noted that getting this information out to the general public is a more difficult task, as there is already a lot of information on social media about diabetes and uptake on some of these resources was lower than we had hoped. This was anticipated to be a challenge, given that most people seeking diabetes resources would be looking for help and support with their self-management rather than research on that issue, and thus our expectations on resource uptake were modest.

#### Considerations prior to the commencement of research

The use of the project name ‘DIPLOMA’ was confusing for us as public contributors, as the acronym bears no meaning with the NHS Diabetes Prevention Programme. Such acronyms used by academics can hinder understanding among public contributors; for example, when PPIE members tried to find out more information about the DIPLOMA project, internet searches brought up information about education courses rather than the NHS Diabetes Prevention Programme. It was reflected that although project acronyms can ease communication internally within the research team, it may not be an accessible shorthand for public contributors, and the wider public, to understand. Going forward, it was discussed that any acronyms and project names could be agreed at the start of the project with the PPIE group who are an integral part of the team and need to be comfortable with using them.

The PPIE group were paid for their involvement throughout DIPLOMA, in line with NIHR guidance [17]. However, one of our PPIE members experienced difficulties in receiving cash reimbursements as this was considered as income by their local Job Centre office, and hence impacted the benefits they were entitled to receive. Alternative arrangements were therefore put in place by the PPIE facilitator to provide payment to this member using vouchers instead. This, however, took considerable time to put in place and the PPIE member received a late payment as a result. Researchers should consider ensuring that they include arrangements in their PPIE strategy for the option to pay public contributors in vouchers, prior to receiving project funding.

#### Involvement with different aspects of the research project

The group have enjoyed being involved in so many aspects of the research, from commenting on study materials (e.g., topic guides, questionnaires) to involvement with the public engagement work. We appreciated how researchers genuinely involved us in every area of the project, as well as the positive feedback on our involvement from the research team, which made our hard work and contributions feel appreciated; an example where the group have worked together towards a common purpose and respected different perspectives [14].

When asked to review study materials, our group appreciated the flexibility of the timings to review documents, especially if some members were unable to meet deadlines due to personal circumstances (e.g., caring responsibilities, health reasons). In line with the inclusive opportunities standard of the recently developed framework [14], providing this flexibility meant that all voices were still heard and provided a more inclusive and considerate approach. Being part of this PPIE group allowed members to contribute to research where we felt we were really making a difference, especially those members who have experience of pre-diabetes and diabetes either personally or within their family. It was felt that comments were taken on board and voices were heard, which felt like ‘true PPIE’ rather than just a tick box exercise.

## Conclusions and recommendations

This commentary has reflected on the PPIE contribution to the DIPLOMA evaluation of the NHS Diabetes Prevention Programme. Based on our learning, we have put together recommendations for other research teams involved in longer-term commissioned research projects like DIPLOMA (see Table 2), in addition to what NIHR already advise as best practice in involving members of the public in research [30]. Many of our reflections correspond with those previously identified in research involving patients and members of the public [6, 16, 31]. However, we have added a unique reflection of PPIE contribution for a longer-term commissioned research evaluation of an NHS service that was already implemented in routine practice, which comes with its own challenges. These reflections and recommendations provide insights to inform future PPIE plans for stakeholders involved in commissioned research evaluations.

**Table 2:** Recommendations for PPIE in commissioned research evaluations of nationally implemented programmes

**Pre-funding of the research project**
Researchers could work with public contributors to agree on a project name that the whole group feel comfortable using, avoiding the use of complicated acronyms where possible Consider alternative remuneration methods for PPIE members (e.g., voucher payments), based on their preferences or potential adverse impact on their income/benefit entitlements
**Start of the research project**
Provide detailed information about the project timelines, roles of the PPIE group, and scope of the PPIE input, to ensure all members are informed at the start of the process on what is expected Be explicit about the type of research that public contributors are getting involved in, for example, distinguishing between research that designs a service and research that evaluates a service Gather ideas from the PPIE group at the start of the project on how they feel they could contribute to the research to ensure that the purpose of public involvement has been jointly agreed
**Throughout the research project**
Group facilitators could plan a schedule for upcoming meetings in advance, to ensure meetings accommodate for everyone’s preferences (including those of the researchers and PPIE members) and to avoid having long breaks in between meetings If there are long breaks between meetings, PPIE members should be kept up to date with developments from the project The involvement of someone who is independent of the research team could collate formal feedback from the PPIE group on a regular basis to provide contributors with more opportunity and scope to feedback on any issues that need addressing throughout the duration of the research Longer project timelines may inevitably result in a turnover of PPIE research facilitators and/or public contributors. Measures should therefore be put in place to ensure handover between group facilitators, inductions for new members, and minimal disruption for the group if this occurs The research team should ensure adequate support is provided to PPIE members, in training and resources, as well as in time, recognising their role as voluntary contributors who have their own commitments outside of the research project
**Resources to consider**
If using remote involvement, facilitators should provide training in how to use video conferencing to ensure all members feel at ease with the technology before meetings. Nonetheless, face-to-face meetings also remain important to allow the development of relationships and trust within the group, especially at the start of their involvement when the group are new to their PPIE role. Thus, if using remote involvement, a hybrid approach with the opportunity for both in-person and online meetings would be beneficial Researchers should consider the extra resources and training that may be required for (a) remote meetings, including the necessary equipment such as a camera for video conferencing, and (b) the use of complex methods used in the research. Such resources and training should be budgeted for in the original funding application

## Supplementary Information

**Additional file 1.** GRIPP2 Short Form Checklist.

## Data Availability

Not applicable.

## Abbreviations

ARC GM: Applied Research Collaboration Greater Manchester
COVID-19: Coronavirus Disease 2019
DIPLOMA: Diabetes Prevention—Long-term Multimethod Assessment
GP: General Practitioner
GRIPP2: Guidance for Reporting Involvement of Patients and the Public
INVOLVE: A national advisory group which promotes and supports greater public involvement in NHS, public health and social care research
NIHR: National Institute for Health and Care Research
NHS: National Health Service
PPIE: Patient and Public Involvement and Engagement
SSC: Study Steering Committee
UK: United Kingdom
