# Supplementary material for: Reflections of patient and public involvement from a commissioned research project evaluating a nationally implemented NHS programme focused on diabetes prevention
Source: Res Involv Engagem. 2023 Jun 14;9:42. doi: 10.1186/s40900-023-00447-0 (PMC10264875; doi:10.1186/s40900-023-00447-0)
Supplement: Supplementary file 1 — Additional file 1. GRIPP2 Short Form Checklist. [file 40900_2023_447_MOESM1_ESM.docx]

**Additional File 1: GRIPP2 Short Form Checklist**

| **Section and topic** | **Item** | **Reported on page No** |
| --- | --- | --- |
| 1. Aim | Report the aim of PPI in the study | 8 |
| 2. Methods | Provide a clear description of the methods used for PPI in the study | 8-10 |
| 3. Study results | Outcomes – report the results of PPI in the study, including both positive and negative outcomes | 10-13 |
| 4. Discussion and conclusions | Outcomes – comment on the extent to which PPI influenced the study overall. Describe positive and negative effects | 13-15 |
| 5. Reflections/critical perspective | Comment critically on the study, reflecting on the things that went well and those that did not, so others can learn from this experience | 15-20 |
